# Correction: Rapid transition to distance learning due to COVID-19: Perceptions of postgraduate dental learners and instructors

**DOI:** 10.1371/journal.pone.0253683

**Published:** 2021-06-17

**Authors:** Fatemeh Amir Rad, Farah Otaki, Zaid Baqain, Nabil Zary, Manal Al-Halabi

The first author’s initials appear incorrectly in the citation. The correct citation is: Amir Rad F, Otaki F, Baqain Z, Zary N, Al-Halabi M (2021) Rapid transition to distance learning due to COVID-19: Perceptions of postgraduate dental learners and instructors. PLoS ONE 16(2): e0246584. https://doi.org/10.1371/journal.pone.0246584

An additional affiliation is missing for the third author. Zaid Baqain is also affiliated with School of Dentistry, University of Jordan, Amman, Jordan.
