# Supplementary material for: Rapid transition to distance learning due to COVID-19: Perceptions of postgraduate dental learners and instructors
Source: PLoS One. 2021 Feb 8;16(2):e0246584. doi: 10.1371/journal.pone.0246584 (PMC7870061; doi:10.1371/journal.pone.0246584)
Supplement: S1 Appendix — (DOCX) [file pone.0246584.s001.docx]

**Evaluating the Learning and Teaching Experience during COVID-19 (Students' Perspective)**

Dear student,

Kindly focus your feedback on the period of learning and teaching as of the recent, unforeseen, and necessary transition to distance learning due to the COVID-19 pandemic. It is each student’s responsibility to provide constructive and thoughtful feedback on their learning experience. Participation remains completely voluntary. Your privacy and the data confidentiality are protected, and no personal identifiers will be recorded.

1. **Which cohort do you belong to?**

- Class of 2022
- Class of 2023
- Class of 2024
- Class of 2025

1. **Please rate the following statements that relate to your learning experience since the transition to distance learning:**

|  | Strongly Disagree | Disagree | Neutral | Agree | Strongly Agree |
| --- | --- | --- | --- | --- | --- |
| The transition to the online environment was clearly explained. |  |  |  |  |  |
| The technology used in the online environment worked effectively. |  |  |  |  |  |
| Adequate opportunities to express my viewpoints and questions were offered to me, during the distance learning. |  |  |  |  |  |
| The online courses’ materials were easy to access. |  |  |  |  |  |
| The online courses’ materials suitably contributed to my learning. |  |  |  |  |  |
| The online courses’ materials available were adequate to meet my learning goals. |  |  |  |  |  |
| Overall, I was satisfied with my distance learning. |  |  |  |  |  |

1. **For students of Classes 2022 and 2023, to what extent did the distance learning prepare you for the upcoming clinical clerkships?**

| 1 Not at all | 2 | 3 | 4 | 5 | 6 | 7 | 8 | 9 | 10  Extremely |
| --- | --- | --- | --- | --- | --- | --- | --- | --- | --- |

1. **The transition to the online environment, in response to the COVID-19, significantly impacted my learning in these courses.**

- Yes
- No

1. **Please elaborate upon your answer to Question 4:**

|  |
| --- |

1. **The transition to the online environment, in response to the COVID-19, significantly impacted the courses’ structure and delivery.**

- Yes
- No

1. **Please elaborate upon your answer to Question 6:**

|  |
| --- |

1. **What were some of the advantages of transitioning to distance learning (e.g., flexible schedule)?**

|  |
| --- |

1. **What were some of the challenges that you faced due to transitioning to distance learning (e.g., lack of face-to-face interactions)?**

|  |
| --- |

1. **Please share your thoughts about the alternative modes of instruction deployed that were particularly supportive of your learning during the COVID 19 pandemic.**

|  |
| --- |

1. **What aspects of those alternative modes of instruction would you like to sustain on the long run (even after returning to regular face-to-face sessions)?**

|  |
| --- |

**Evaluating the Learning and Teaching Experience during COVID-19 (Instructors' Perspective)**

Kindly focus your feedback on the period of learning and teaching as of the recent, unforeseen, and necessary transition to distance learning due to the COVID-19 pandemic. Participation remains completely voluntary. Your privacy and the data confidentiality are protected, and no personal identifiers will be recorded.

1. **Please rate the following statements that relate to your learning experience since the transition to distance learning:**

|  | Strongly Disagree | Disagree | Neutral | Agree | Strongly Agree |
| --- | --- | --- | --- | --- | --- |
| The transition to the online environment was clearly explained. |  |  |  |  |  |
| The technology used in the online environment worked effectively. |  |  |  |  |  |
| The University provided me with adequate and timely support throughout the distance teaching. |  |  |  |  |  |
| The courses’ content and materials were easy to share online. |  |  |  |  |  |
| The case-based scenarios, used throughout the distance learning, was helpful, in my opinion, to develop the students’ clinical knowledge. |  |  |  |  |  |
| Overall, I was satisfied with the distance learning. |  |  |  |  |  |

1. **The transition to the online environment, in response to the COVID-19, significantly impacted my teaching in these courses.**

- Yes
- No

1. **Please elaborate upon your answer to Question 2:**

|  |
| --- |

1. **The transition to the online environment, in response to the COVID-19, significantly impacted the courses’ structure and delivery.**

- Yes
- No

1. **Please elaborate upon your answer to Question 4:**

|  |
| --- |

1. **What were some of the advantages of transitioning to distance learning?**

|  |
| --- |

1. **What were some of the challenges that you faced due to transitioning to distance learning?**

|  |
| --- |

1. **Please reflect upon the alternative modes of instruction deployed that were particularly supportive, in your opinion, for the students’ distance learning.**

|  |
| --- |

1. **What aspects of those alternative modes of instruction would you like to sustain on the long run (even after returning to regular face-to-face sessions)?**

|  |
| --- |
